# Supplementary material for: Two single-point mutations in Ankyrin Repeat one drastically change the threshold temperature of TRPV1
Source: Nat Commun. 2023 May 11;14:2415. doi: 10.1038/s41467-023-38051-1 (PMC10175561; doi:10.1038/s41467-023-38051-1)
Supplement: Supplementary file 1 — Supplementary Information [file 41467_2023_38051_MOESM1_ESM.pdf]

Supplementary Fig1

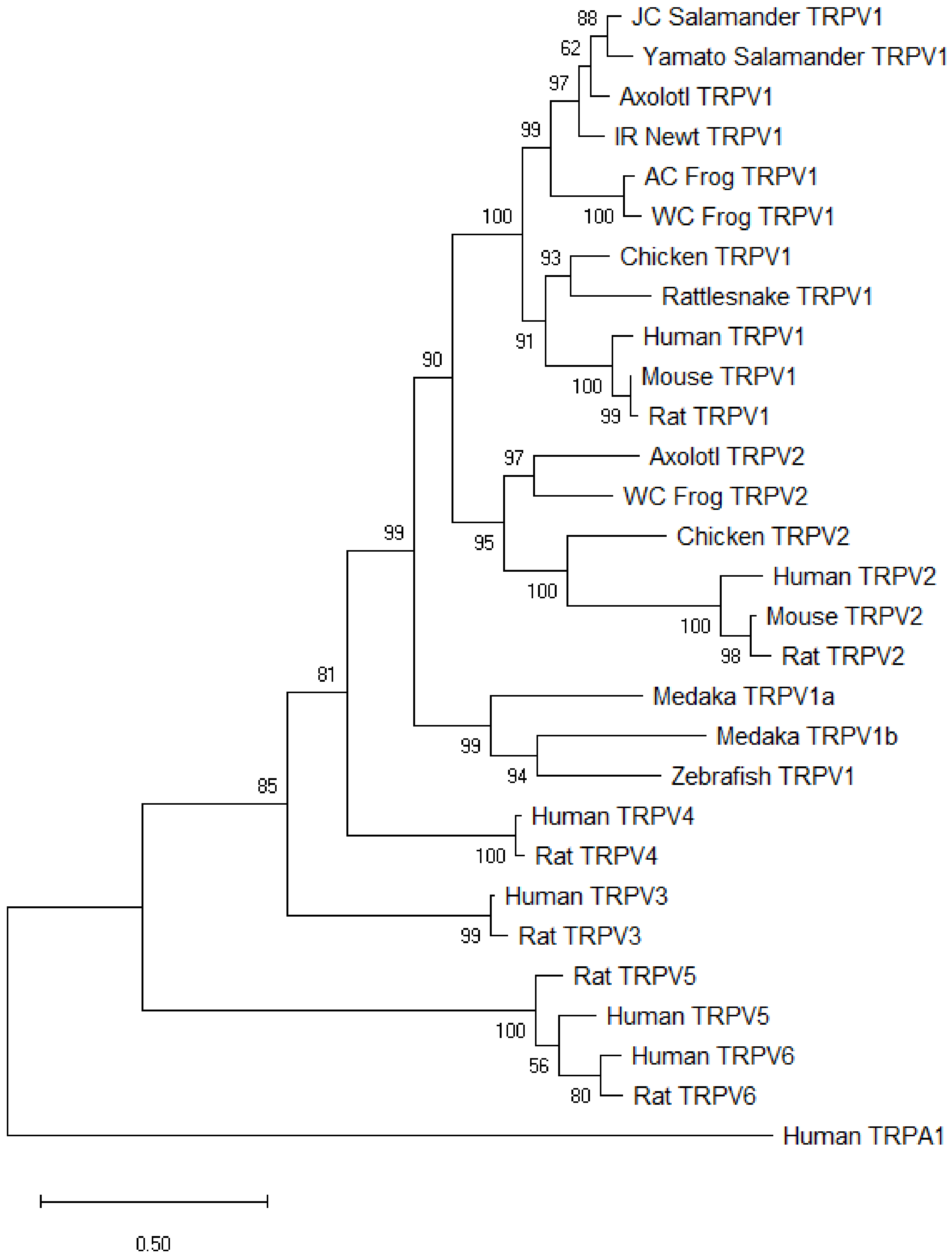

Supplementary Fig2

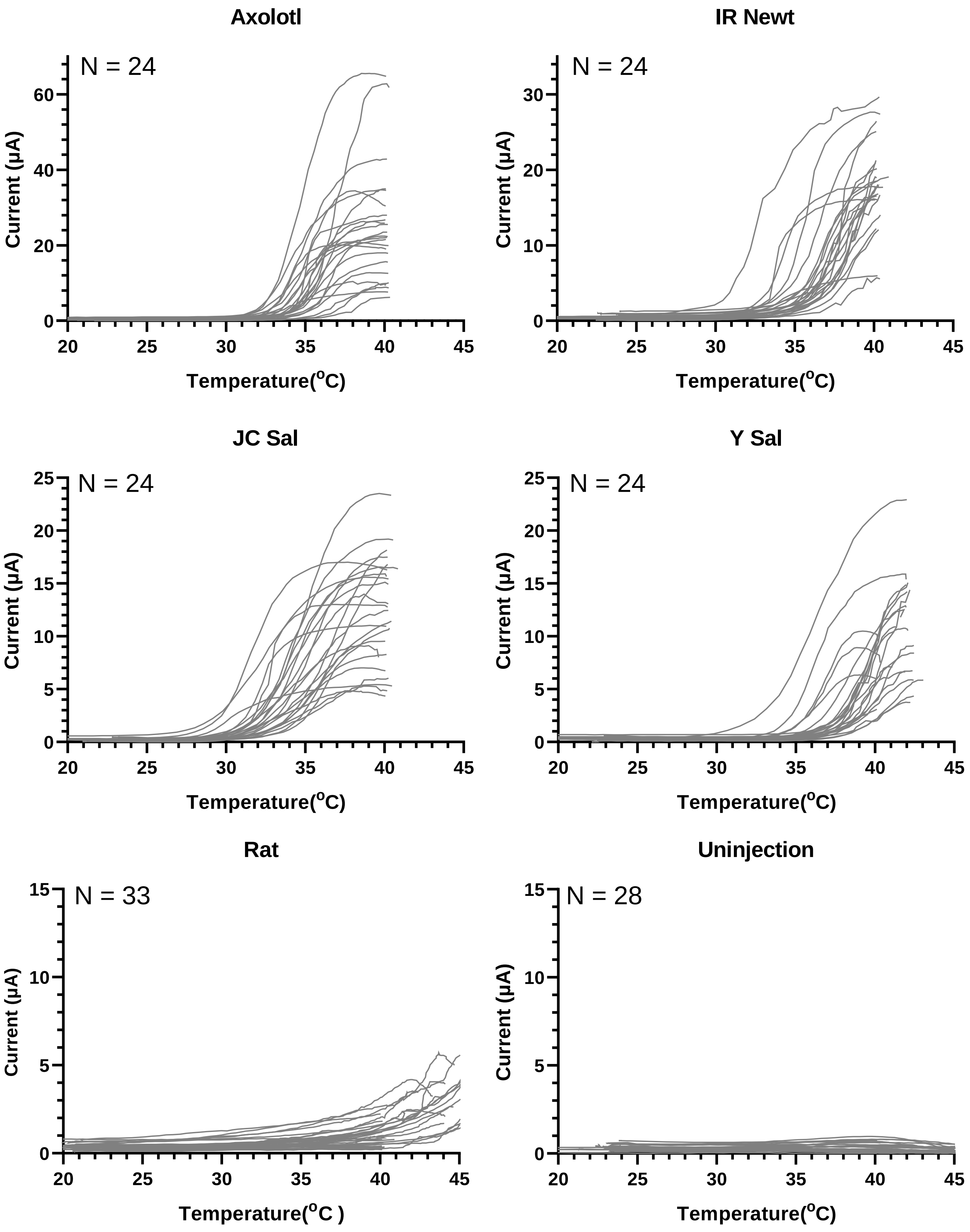

Supplementary Fig3

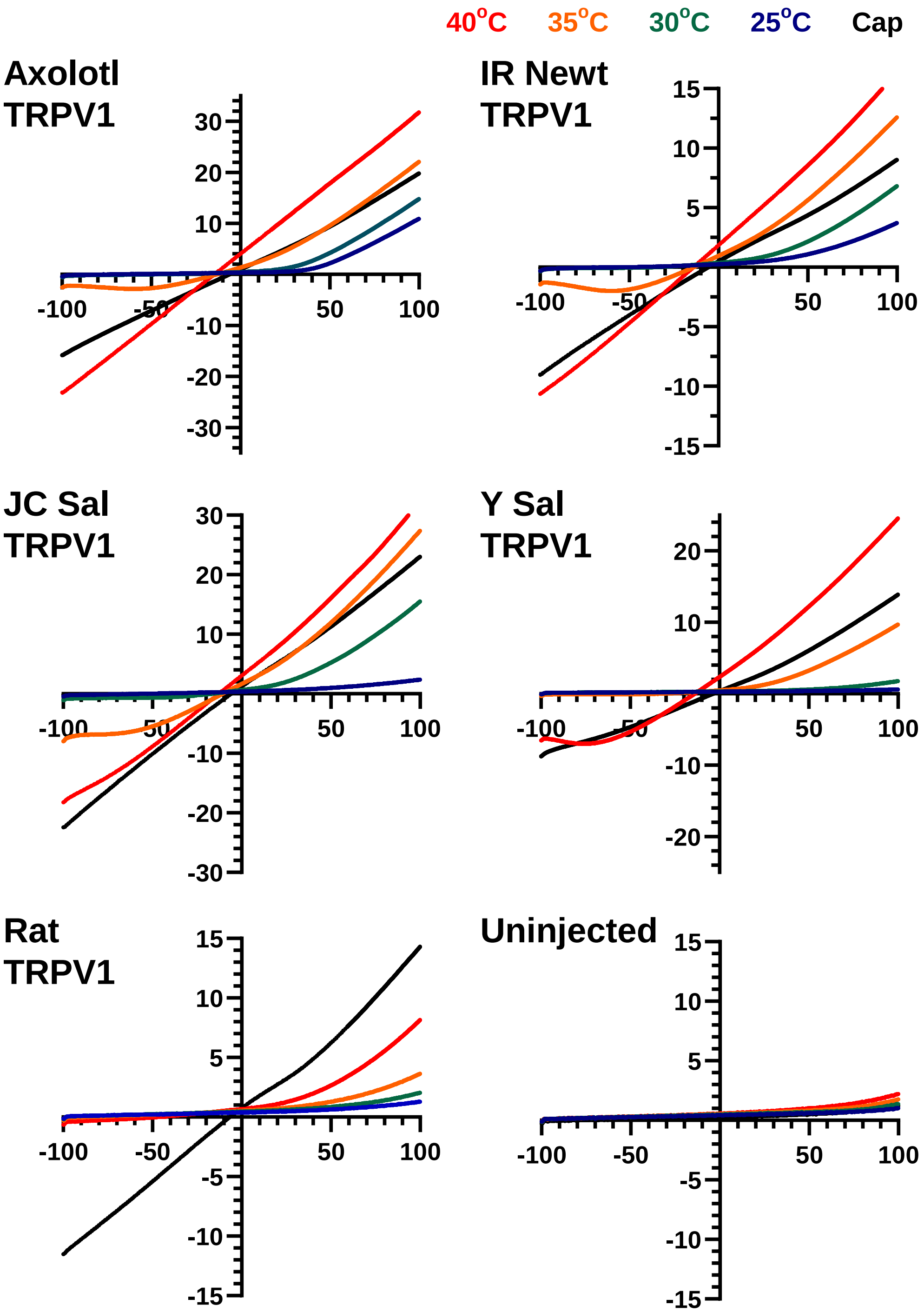

Supplementary Fig4

	S124
K	-0.97
G	-0.95
R	-0.39
N	-0.39
H	-0.31
F	-0.22
Q	-0.22
C	-0.09
S	0.00
L	0.04
Y	0.06
M	0.14
A	0.15
W	0.24
D	0.34
E	0.36
T	0.75
V	1.36
I	2.62
P	5.59

	Q188
E	-1.55
P	-0.97
M	-0.52
K	-0.41
F	-0.38
Y	-0.34
L	-0.34
V	-0.25
W	-0.23
I	-0.19
C	-0.17
T	-0.15
D	-0.13
R	-0.08
A	-0.02
Q	0.00
S	0.08
H	0.13
G	0.13
N	0.20

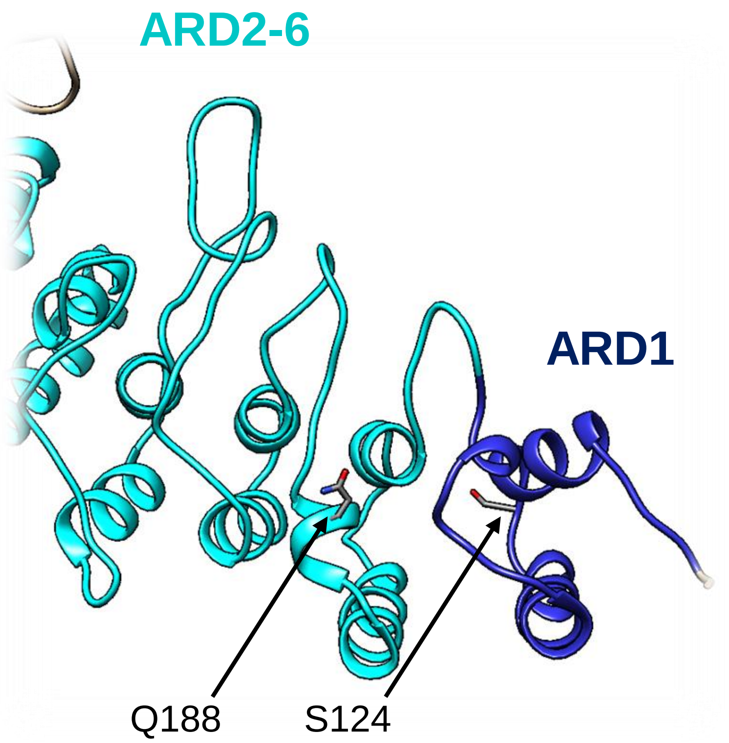

Supplementary Figure Legends

Supplementary Figure 1. Phylogenetic tree of TRPV1-6

Amino acid sequences of ANK1-TRPbox of TRPV1s from tailed amphibians, mammals, birds, reptiles, frogs, and fish, TRPV2s-6s from mammals, and human TRPA1 were used for a maximum likelihood analysis.

Supplementary Figure 2. Heat response of TRPV1 from four urodelans and rat

Urodelan or rat TRPV1 was expressed in *Xenopus* oocytes and the heat-induced response was examined by the two-electrode voltage clamp method. Heat responses at −80mV for the individual oocytes expressing each TRPV1 are indicated.

Supplementary Figure 3. I-V relationship for heat response of tailed amphibian TRPV1

Urodelan or rat TRPV1 was expressed in *Xenopus* oocytes. The capsaicin- or heat-induced response was examined by the two-electrode voltage clamp method. The I–V relationship for the heat response at the indicated temperature and for the capsaicin-response (urodelans: 50 μM, rat: 4 μM) are shown.

Supplementary Figure 4. The mutational folding energy changes at 124S and 188Q of rat TRPV1

Based on homology modeling of rTRPV1 described in Fig. 9, the mutational folding energy changes ($\Delta\Delta G$) at 124S and 188Q were evaluated with FoldX. The positions of 124S and 188Q in the structure of the N-terminus are shown.

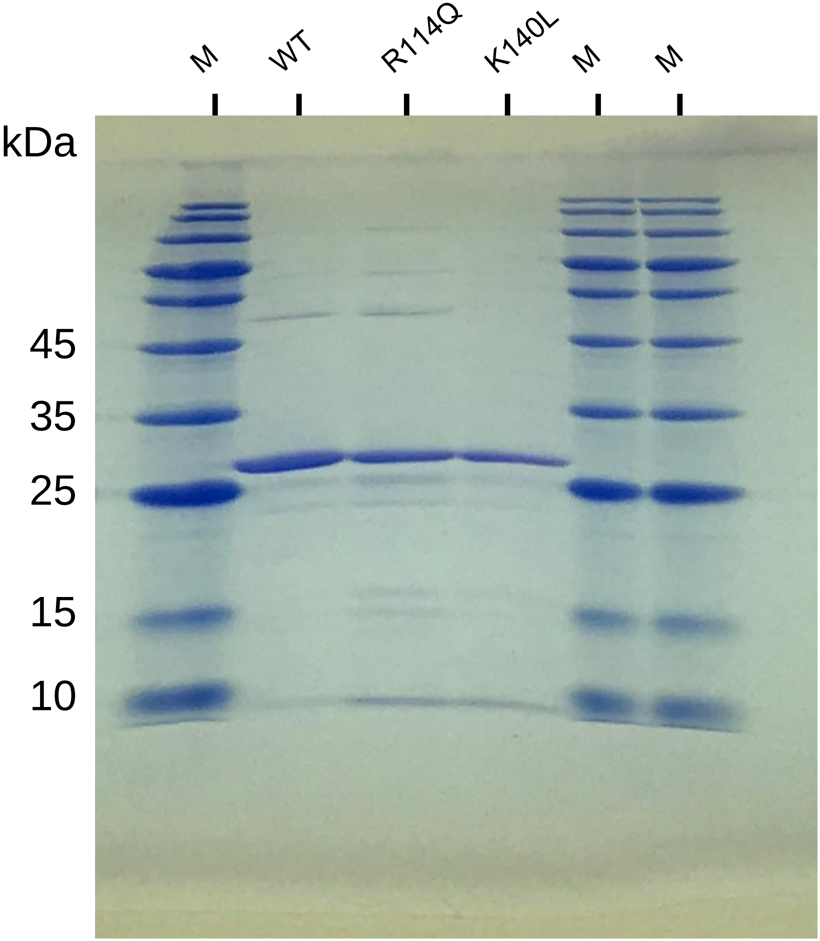

The proteins of the N-terminus (100-362aa) from wild rTRPV1 and two mutants (R114Q, L140L) were purified and analyzed by SDS-PAGE.
