# Supplementary material for: Two single-point mutations in Ankyrin Repeat one drastically change the threshold temperature of TRPV1
Source: Nat Commun. 2023 May 11;14:2415. doi: 10.1038/s41467-023-38051-1 (PMC10175561; doi:10.1038/s41467-023-38051-1)
Supplement: Supplementary file 3 — Reporting Summary [file 41467_2023_38051_MOESM3_ESM.pdf]

nature portfolio

Corresponding author(s): Osamu Saitoh

Last updated by author(s): Mar 31, 2023

Reporting Summary

Nature Portfolio wishes to improve the reproducibility of the work that we publish. This form provides structure for consistency and transparency in reporting. For further information on Nature Portfolio policies, see our Editorial Policies and the Editorial Policy Checklist.

Statistics

For all statistical analyses, confirm that the following items are present in the figure legend, table legend, main text, or Methods section.

n/a	Confirmed	
☐	☒	The exact sample size (n) for each experimental group/condition, given as a discrete number and unit of measurement
☒	☐	A statement on whether measurements were taken from distinct samples or whether the same sample was measured repeatedly
☐	☒	The statistical test(s) used AND whether they are one- or two-sided<br>*Only common tests should be described solely by name; describe more complex techniques in the Methods section.*
☒	☐	A description of all covariates tested
☐	☒	A description of any assumptions or corrections, such as tests of normality and adjustment for multiple comparisons
☐	☒	A full description of the statistical parameters including central tendency (e.g. means) or other basic estimates (e.g. regression coefficient) AND variation (e.g. standard deviation) or associated estimates of uncertainty (e.g. confidence intervals)
☐	☒	For null hypothesis testing, the test statistic (e.g. F, t, r) with confidence intervals, effect sizes, degrees of freedom and P value noted<br>*Give P values as exact values whenever suitable.*
☒	☐	For Bayesian analysis, information on the choice of priors and Markov chain Monte Carlo settings
☒	☐	For hierarchical and complex designs, identification of the appropriate level for tests and full reporting of outcomes
☐	☒	Estimates of effect sizes (e.g. Cohen's d, Pearson's r), indicating how they were calculated

Our web collection on statistics for biologists contains articles on many of the points above.

Software and code

Policy information about availability of computer code

Data collection	No software was used.
Data analysis	pClamp 9, MODELER 10.1, PHENIX 1.19.2, MOLPROBITY 4.5, FOLDX 4

For manuscripts utilizing custom algorithms or software that are central to the research but not yet described in published literature, software must be made available to editors and reviewers. We strongly encourage code deposition in a community repository (e.g. GitHub). See the Nature Portfolio guidelines for submitting code & software for further information.

Data

Policy information about availability of data

All manuscripts must include a data availability statement. This statement should provide the following information, where applicable:

- Accession codes, unique identifiers, or web links for publicly available datasets
- A description of any restrictions on data availability
- For clinical datasets or third party data, please ensure that the statement adheres to our policy

Data supporting the findings of this work are available within the paper and its Supplementary Information files. The source data underlying figures are provided as two Source Data files. Sequencing data that support the findings of this study have been deposited in the DNA Data Bank under the accession number LC728477 (IR Newt TRPV1), LC728478 (Y Sal TRPV1), and LC728479 (JC Sal TRPV1).

Human research participants

Policy information about studies involving human research participants and Sex and Gender in Research.

Reporting on sex and gender	N/A
Population characteristics	N/A
Recruitment	N/A
Ethics oversight	N/A

Note that full information on the approval of the study protocol must also be provided in the manuscript.

Field-specific reporting

Please select the one below that is the best fit for your research. If you are not sure, read the appropriate sections before making your selection.

☒ Life sciences ☐ Behavioural & social sciences ☐ Ecological, evolutionary & environmental sciences

For a reference copy of the document with all sections, see nature.com/documents/nr-reporting-summary-flat.pdf

Life sciences study design

All studies must disclose on these points even when the disclosure is negative.

Sample size	Most experiments were performed three or more independent times. Sample size in all experiments were estimated based in previous published experiments. At least three or more animals were used in animal experiment and at least four or more cells were used in cell physiology experiments to obtain statistical analysis.
Data exclusions	No data was excluded from the analysis.
Replication	All measurements repeated twice or more, and repetitions were all successful
Randomization	Randomly selected samples and animals were allocated into experimental groups.
Blinding	Since results and phenotypes of animals were so obvious, the blinding was unnecessary.

Reporting for specific materials, systems and methods

We require information from authors about some types of materials, experimental systems and methods used in many studies. Here, indicate whether each material, system or method listed is relevant to your study. If you are not sure if a list item applies to your research, read the appropriate section before selecting a response.

Materials & experimental systems

n/a	Involved in the study	
☒	☐	Antibodies
☐	☒	Eukaryotic cell lines
☒	☐	Palaeontology and archaeology
☐	☒	Animals and other organisms
☒	☐	Clinical data
☒	☐	Dual use research of concern

Methods

n/a	Involved in the study	
☒	☐	ChIP-seq
☒	☐	Flow cytometry
☒	☐	MRI-based neuroimaging

Eukaryotic cell lines

Policy information about cell lines and Sex and Gender in Research

Cell line source(s)	HEK293T cell (human embryonic kidney cell line), established by Professor David Baltimore, is used in this study. The HEK293T cell was gifted from Dr. Masaki Iizuka (in Nippon Boehringer Ingelheim Co. Ltd. Pharma Research Institute) to Dr. Yoshihiro Kubo.

Authentication	None of the cell line used were authenticated.
Mycoplasma contamination	Negative.
Commonly misidentified lines (See ICLAC register)	The study does not use misidentified lines.

Animals and other research organisms

Policy information about studies involving animals; ARRIVE guidelines recommended for reporting animal research, and Sex and Gender in Research

Laboratory animals	Two or three years old animals of Xenopus laevis and Pleurodeles waltl were used.
Wild animals	Two years old adult animals of Hynobius vandenburghi and one or two year old larvae of Onychodactylus japonicus were collected with a net and transported in the tank filled with clean fresh water. After the study, they were returned to the same place where they were collected. One or two animals were sacrificed to isolate essential tissue samples by deep anesthesia with ice-cold water or MS222 (an anesthetic and euthanasia agent for aquatic animals).
Reporting on sex	Since there is only limited information, we could not determine sex of tailed amphibians.
Field-collected samples	We are careful to keep the animals in comfortable sized and temperature-controlled tanks (which also varies depending on the species' size and habits, especially for temperature, 25°C for Hynobius vandenburghi, 4°C for Onychodactylus japonicus).
Ethics oversight	Animal experiments were approved by the Animal Experiment Committee of Nagahama Institute of Bio-Science and Technology and were performed based on their guidelines.

Note that full information on the approval of the study protocol must also be provided in the manuscript.
